# Medial Tibial Stress Syndrome: Muscles Located at the Site of Pain

**DOI:** 10.1155/2016/7097489

**Published:** 2016-03-15

**Authors:** Ato Ampomah Brown

**Affiliations:** Department of Anatomy, School of Medical Sciences, University of Cape Coast, Cape Coast, Ghana

## Abstract

*Objective*. The purpose of this study was to examine the relationship between the location of the MTSS pain (posteromedial border of tibia) and the muscles that originate from that site.* Method*. The study was conducted in the Department of Anatomy of the School of Medical Sciences, University of Cape Coast, and involved the use of 22 cadaveric legs (9 paired and 4 unpaired) from 11 males and 2 females.* Findings*. The structures that were thus observed to attach directly to the posteromedial border of the tibia were the soleus, the flexor digitorum longus, and the deep crural fascia. The soleus and flexor digitorum longus muscles were observed to attach directly to the posteromedial border of the tibia. The tibialis posterior muscle had no attachment to this site.* Conclusion*. The findings of this study suggest that if traction is the cause of MTSS then soleus and the flexor digitorum muscles and not the tibialis posterior muscle are the likely cause of MTSS.

## 1. Introduction

The medial tibial stress syndrome (MTSS) also known as shin splints or medial tibial traction periostitis is a common and often debilitating overuse injury of the lower leg associated with running and walking activities and is mostly seen among athletes, military personnel, and recreational sports participants [1, 2]. Incidence in this population is between 4% and 35% [3] and it accounts for between 13.2% and 17.3% of all running injuries [1]. MTSS is the most common exercise induced leg pain; most people who suffer from it complain of pain at the onset of exercise that is felt along the posteromedial border of the tibia, usually in the middle or distal third of the bone [1, 3, 4]. Initially the pain is experienced at the onset of activity and subsides with continued exercise, but later on the pain may persist even during activity [3].

Although the aetiology of MTSS is currently unclear several theories have been put forth to explain this phenomenon. These include (1) repeated bending or bowing of the tibia [3, 5] and (2) traction of the tibial periosteum [6].

Studies have shown that repeated bending causes adaptation of the tibia, predominantly at the site where bending forces are greatest which is approximately at the junction of the middle and distal thirds where the pain from MTSS is usually felt [3, 7, 8].

The traction phenomenon states that repeated traction on the periosteum by fibres of muscles that attaches along the length of the medial border of the tibia [3, 4] and even possibly by the deep crural fascia, which also attaches to the same location, may possibly be responsible for symptoms of MTSS [9–11].

Traditionally the tibialis posterior (TP) muscle has been thought to be the source of this traction; other studies however have implicated the soleus and the flexor digitorum longus (FDL) muscles [4, 9, 12–15].

Though other investigators have researched on this subject [9, 12] there appears however to be no research that has focussed on the West African population. The purpose of this study is therefore to examine the relationship between the location of the MTSS pain (posteromedial border of tibia) and the muscles that originate from that site in Ghanaians.

## 2. Materials and Method

The study was conducted in the Department of Anatomy of the School of Medical Sciences, University of Cape Coast. Twenty-two cadaveric legs (9 paired and 4 unpaired) from 11 males and 2 females were used in the study. These cadavers had been preserved by embalming them with 10% formaldehyde solution. The skin and subcutaneous tissue were dissected off each leg. The two heads of the gastrocnemius were transected and the muscle was reflected inferiorly to expose the underlying structures, the ligaments and tendons that attach to the medial aspect of the knee were removed, and the joint capsule was opened and the medial meniscus removed to expose the superior articular surface of the media condyle of the tibia. The flexor retinaculum of the ankle and the deltoid (medial) ligament of the ankle were also removed to expose the tip of the medial malleolus. The length of the tibia was then measured using anthropometric calipers from the middle of tibial plateau proximally to the tip of the medial malleolus distally. The muscles and structures that attach to the posteromedial border of the tibia were noted. The distances from the tip of the medial malleolus to the distal (superomost) and proximal (inferomost) attachment sites of the muscles that attach to the posteromedial border of the tibia were measured to the nearest 0.1 cm. All measurements were then converted into percentages of the length of their respective tibia bones in order to make comparisons between bones.

The posteromedial aspect of the tibia was also divided into 3 equal segments proximally to distally, and the segments were named the proximal third, middle third, and distal third, respectively. This allowed for the attachment of each muscle to be placed in one or more of these segments.

## 3. Results

In this study the FDL muscle was found to be attached to the medial part of posterior surface of tibia inferior to soleal line, the soleus muscle attached to the posterior surface of proximal part of fibula, soleal line, and the posteromedial aspect of the tibia, while the TP muscle was found to attach to the posterior surface of interosseous membrane, the lateral aspect of the posterior surface of the tibia, and the medial part of the posterior fibular surface.

The deep crural fascia was observed to attach to the proximal 2/3rd of the medial border of the tibia.

## 4. Discussion

**Figure 1:**
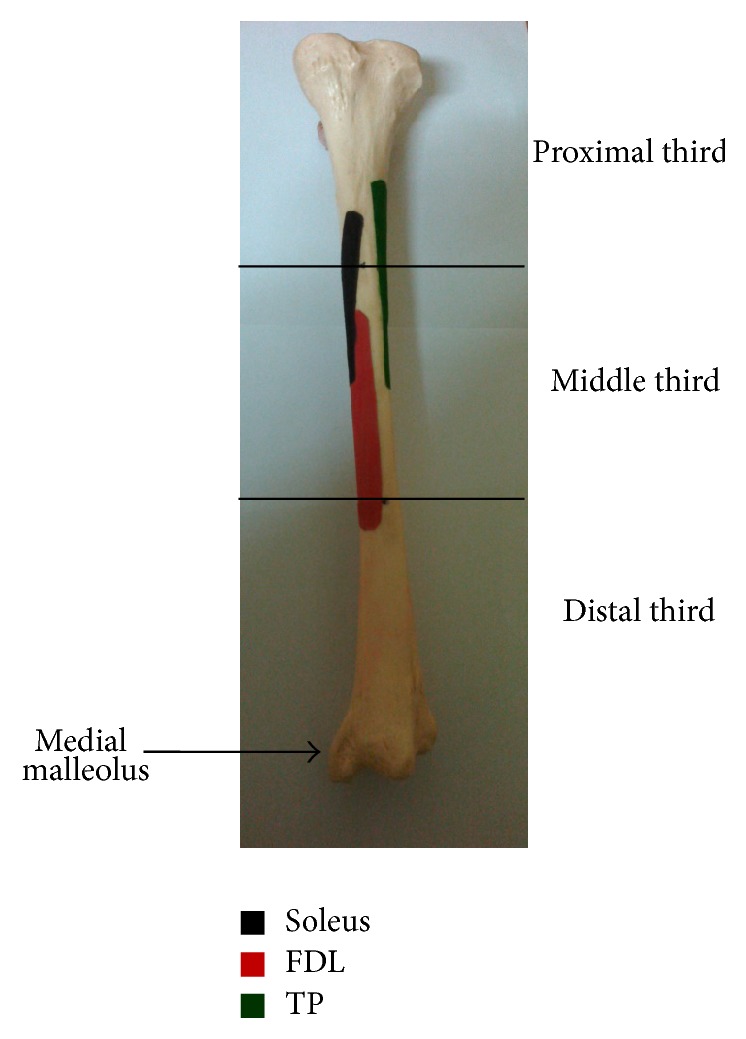
Site of muscle attachment on posterior aspect of the left tibia bone.

In this study it was observed that the FDL, soleus, and the TP all had attachments similar to those described in anatomy text books. The muscle fibres found to attach directly to the posteromedial border of the tibia were the soleus and the FDL. The TP muscle did not have any attachment on the posteromedial border of the tibia (Figure 1). These findings are similar to those of Michael and Holder [12] as well as Beck and Osternig [9] but contrary to the findings of Saxena et al. [16]. The deep crural fascia was also attached to the posteromedial border of the tibia given some credence to the postulation that it may play a role in the aetiology of MTSS.

From Table 1 it is observed that the average proximal and distal attachments of the soleus were found to be 49.5% and 72.5% of the length of the tibia from the medial malleolus, respectively, while those of the FDL were observed to be 33.3% and 59.6%, respectively. Findings are similar to those reported by Beck and Osternig [9].

From the literature the pain of MTSS is described as being located along the posteromedial border of the tibia, usually in the middle to distal thirds [1–3], but it has also been reported to occur in the proximal third of tibia.

The data in Table 2 shows that in 12 of the cadaveric legs the fibres of the FDL muscle were attached to middle and distal third of the tibia, while the remaining 11 legs had their FDL muscle fibres situated only in the middle third tibia. Of the 12 that had fibres overlapping the middle and distal third, 88.1% of the attachment length fell within the middle third of the tibia (Table 3); thus, in all specimen the FDL was mainly attached to the middle third, the area where the MTSS pain is most commonly felt.

With the exception of one specimen all the fibres of the soleus muscle extended from the middle third into the proximal third (Table 2). Similar to the FDL muscle the majority of muscle fibres (72.7%) were also found attached to the middle third of the tibia giving credence to suggestion of other researchers that the FDL and soleus muscles may play a part in the aetiology of MTSS [9, 12]. The extension of muscles fibres of the soleus from the middle third into the proximal third and the extension of some muscle fibres of the FDL into the distal thirds from the middle third may further explain why MTSS symptoms are sometimes experienced in the proximal as well as the distal portions of the tibia.

In 2009 Murley et al. [17] found that individuals with a pronated foot, which is an intrinsic risk factor for developing MTSS [18–21], had greater Electromyography (EMG) amplitudes for the soleus muscle during certain phases of the gait cycle, suggesting a possibility of increased contraction and traction on the underlying periosteum in these individuals.

Wolff's Law, which describes how bone tissue adapts in response to the mechanical forces that are applied to it, [22, 23] has been used to explain how repeated bending of the tibia may be the possible cause of MTSS. It has been found that bending moments generate electrical potentials across bone tissue. This piezoelectric effect is thought to influence the resorption and deposition of bone tissue [23]. In the tibia bone the site of greatest bending occurs at the junction of the middle and distal thirds of its shaft [3]. It is this observation that has made some researchers conclude that the piezoelectric effect generated at this location during running may possibly result in a net resorption of bone tissue resulting in the pain experienced in MTSS [2].

The literature describes a variety of regimens in the treatment of MTSS; these include calf muscle training, using antipronation insoles, massage, electrotherapy, acupuncture, and surgery [3].

If traction is the cause of MTSS then modalities like rest from activities that will cause excessive traction, stretching, and the use of orthoses that correct excessive pronation (pronation causes of excessive contraction and traction on the periosteum) should be considered.

Though the aetiology is still being debated this researcher is of the opinion that if traction is cause of MTSS then the FDL and soleus muscles and not the tibialis posterior muscle are the most likely source of this traction and hence likely aetiological factors in MTSS.

## Figures and Tables

**Table 1:** The mean distance of the attachment of muscle fibres from the medial malleolus of tibia.

Name of muscle	Proximal (Inferomost)	Distal (Superomost)	Mean distance of muscle fibre attachment to tibia
FDL	33.3 ± 3.4	59.6 ± 2.7	26.3 ± 4.3
Soleus	49.5 ± 3.4	72.5 ± 1.8	23.0 ± 4.1

Note: all values are percentages of the length of the tibia bone ± standard deviation.

**Table 2:** Number of specimens that have muscle fibres attachment in the different segments of posteromedial border of the tibia.

Name of muscle	Proximal third	Middle third	Distal third
	*n* (%)	*n* (%)	*n* (%)
FDL	0 (0.0)	23 (100)	12 (52.2)
Soleus	22 (95.7)	23 (100)	0 (0.0)

**Table 3:** Mean percentage distribution of length of attachment for muscles located in different segments of the posteromedial border of tibia.

Name of muscle	Proximal third	Middle third	Distal third
FDL	0%	88.1%	11.9%
Soleus	27.3%	72.7%	0%
